# Some Account of a Case of Softening of the Heart and Emphysema of the Lungs, with the Morbid Appearances after Death

**Published:** 1828-10

**Authors:** 

**Affiliations:** Cincinnati


ART. III.—*Some account of a fatal case of Softening of the Heart with apparent Emphysema of the Lungs. By the* EDITOR.

On the 30th of July, 1828, I was requested to visit Mr. R. B. and continued my attendance upon him till the 1st of September, when he died.

The patient was 67 years of age, a native of England, by occupation a cordwainer, of sober and industrious habits, in size above the ordinary standard, rather corpulent, but of an athletic temperament.

For more than half his life he had been afflicted, on both arms, with an eruptive disease, which seems to have been a variety of dry psoriasis. About 18 months before his death, it left those parts, and attacked his head and face. The itching in both cases was, occasionally, intolerable; and between the disease and the rubbing of the parts, which it occasioned, he lost all the hair of both his eyebrows; and the affected skin assumed a sooty complexion. Six or eight weeks before I saw him, it abated on the face, and fell down to his chest, from which it disappeared before his death. Soon after attacking this part, he experienced occasional paroxysms of pain in the region of the heart. When I was called in, at the time above mentioned, he was confined to his bed.

His respiration was somewhat embarrassed, but he had little or no cough; his pulse was intermitting, rather increased in frequency—but not very strong; he had a slight swelling of the abdomen, and considerable œdema of the feet and legs, which were habitually cold. His tongue was clean, and his bowels easily moved; but his appetite was gone, and he frequently vomited. His spirits were habitually depressed, and he often shed tears. In the day he slept a little; but his morbid vigilance and restlessness at night were distressing in the highest degree, and accompanied by a kind of delirium. His chest, under percussion, sounded well; and, through the stethoscope, a *feeble* respiratory murmur could be heard over every part. On resorting to the instrument with a view to the movements of the heart, I could distinctly perceive its intermissions, but neither its sound, impulse, nor volume seemed to be remarkable—they were indeed rather weak. He could lie on either side, and on his back, in a horizontal posture, without difficulty.

I was undecided as to the diagnosis of his malady; but on the whole, presumed it to be an original hepatic disorder occasioning the eruption, sooty complexion, nausea, and dropsical effusions. The irregular pulse I supposed to be caused by a hydro-pericardium.

He had been treated by his physician with stimulants and narcotics of all kinds, without the least benefit. Admonished by this, and guided by the existing symptoms, I put him on the use of the blue pill, a compound infusion of digitalis, and a hot salt bath, with frictions to his feet and legs. Under this treatment his bowels were kept regular, the temperature of his lower extremities was nearly restored, and a diuresis occurred, which was attended with the disappearance of all tumefaction from the abdomen and lower extremities. Such effects inspired a transient hope, that he might be cured; but I soon discovered that no further amendment was taking place; that his paroxysms of vomiting occurred oftener; that he was undergoing a rapid emaciation; that his pulse became daily more irregular; that his hypochondraism was transformed into a kind of melancholic delirium; under which he would rise from his bed, and, fancying himself from home, endeavour to escape from his room; finally, that his morbid watchfulness, gave place to the opposite condition of taciturnity and stupor. It was now sufficiently obvious,that he laboured under a malady of the heart; but of what kind I was unable to decide. I bled him once. His blood was sizy, but no mitigation of the symptoms followed. A blister to the epigastrium did no good, either to the heart or stomach; cupping over the former organ was attempted; but by this time he had lost fat, till the integuments were so loose, as to fill the vacuum in the cups, and prevent the escape of blood. Finally, recourse was had to the sulphate of morphia, the bark and other tonics and stimulants, but they produced no sensible effects. In the closing stage of the malady, his mental faculties suffered still greater impairment, and his dyspnœa became much more distressing. He was long *in articulo mortis*, and exhibited an agitation of the heart, lungs, and muscles of respiration, which it was distressing to witness. The diaphragm especially, seemed to be strongly convulsed.

To abridge this narrative of symptoms, I would select the following as characteristic and constant:—Habitually intermitting pulse—occasional vomiting—epigastric anxiety—low spirits—debility and confusion of mind, and morbid vigilance succeeded by coma. Not long before his death he was taken out in a carriage, but was comatose on the jaunt.

### Dissection.

Permission was granted to examine the contents of the chest and abdomen; but circumstances limited us to a period of time, too short, for a perfect search into their condition.

On proceeding to open the thorax, the cartilages of several ribs, on each side, were found in a state of ossification, and required the use of the saw.

The lungs did not collapse, and *seemed* to be universally emphysematous. As far as examined, they were neither tuberculous nor hepatized. No pleuretic adhesions existed; but in examining for them, I was surprised to find a quantity of dark fluid blood, in each side of the chest. In the right, it scarcely amounted to an ounce, but in the left there was, by estimate, more than two ounces.

The heart was enveloped in an extraordinary quantity of fat. It was of the common size; but far from the ordinary shape, its apex being enlarged and tuberous. It was evident from external inspection, that the pericardium contained no water; and on cutting through that membrane, with a view to turn out the heart, it was found adhering to that organ throughout its whole extent; indicating a universal pericarditis. At its junction with the pleura of the diaphragm, the effects of inflammation were equally obvious, and extended to some distance on the surrounding parts of that organ. At the point of union between the two, the pericardium had given way, and about the aperture there was a quantity of red, pulverulent, pulpy matter, which was the muscular substance of the heart, transformed by disease. In cutting upwards from this place at right angles to the septum, so as to divide the organ into halves, the whole extent of the diseased structure was exposed to view. For more than an inch, the parieties of each ventricle, together with the partition, had lost its fibrous texture, was softened, and flaky or laminated, so that the finger passed readily through it, in all directions. The transformation was greatest at the apex, indicating that it had commenced in that part. Its termination above was not pointed out by any line of demarkation, and the morbid action which occasioned it had continued to advance upwards to the time of death. The columnæ carneæ had experienced the fate of the parieties, but beyond the limits of the disorganized part, they were entire. Both they and the parieties, however, were paler, and more relaxed than in the healthy heart. The valves were free from degeneracy.

The immediate cause of the death of the patient was now made obvious. As long as the disorganization had not yet extended through the parieties, or the pericardium remained entire, the circulation could be maintained, but when the læsion became so great as to suffer blood to escape into the cavities of the pleura, the dyspnœa would of course increase; at the same time the debility of the organ would prevent it from injecting itself, the lungs, brain, and spinal marrow, with the blood necessary to their support; while that which they received was undoubtedly, from the emphysema of the lungs, by no means sufficiently æreated, to enable it to sustain the energies of the system.

In the partial examination which was permitted, of the abdominal viscera, they were found to be healthy. Of the whole, the liver was examined with the greatest care, and contrary to my expectation, was unaltered in size, colour, and texture.

*Cincinnati, October 1st,* 1828.

